# Integrate-and-Fire Neuron Circuit Without External Bias Voltages

**DOI:** 10.3389/fnins.2021.644604

**Published:** 2021-03-24

**Authors:** Young-Soo Park, Sola Woo, Doohyeok Lim, Kyoungah Cho, Sangsig Kim

**Affiliations:** Department of Electrical Engineering, Korea University, Seoul, South Korea

**Keywords:** *p*-*n*-*p*-*n* diode, technology computer-aided design simulation, latch-up phenomenon, integrate-and-fire neuron, spiking neural networks, absence of external bias lines

## Abstract

In this study, we propose an integrate-and-fire (I&F) neuron circuit using a *p-n*-*p-n* diode that utilizes a latch-up phenomenon and investigate the I&F operation without external bias voltages using mixed-mode technology computer-aided design (TCAD) simulations. The neuron circuit composed of one *p-n-p-n* diode, three MOSFETs, and a capacitor operates with no external bias lines, and its I&F operation has an energy consumption of 0.59 fJ with an energy efficiency of 96.3% per spike. The presented neuron circuit is superior in terms of structural simplicity, number of external bias lines, and energy efficiency in comparison with that constructed with only MOSFETs. Moreover, the neuron circuit exhibits the features of controlling the firing frequency through the amplitude and time width of the synaptic pulse despite of the reduced number of the components and no external bias lines.

## Introduction

Neuromorphic computing architectures mimicking the human brain have been used to perform pattern recognition, classification, and perception to overcome the crucial issue of power consumption faced by Von-Neumann computing architectures while processing complex data and information (Chu et al., 2014; Merolla et al., 2014; Srinivasan et al., 2017). Despite their advantages over Von-Neumann computing architectures in terms of energy efficiency, neuron circuits, driven by spiking neural networks (SNNs), still need more power for their integrate-and-fire (I&F) operations than biological neurons (Choi et al., 2018). For most neuron circuits, particularly those using complementary metal-oxide semiconductor (CMOS), feedback field-effect-transistor (FBFET), and floating gate FET (FGFET) (Indiveri et al., 2006; Kornijcuk et al., 2016; Choi et al., 2018; Kwon et al., 2018; Kim et al., 2019; Wang and Khan, 2019; Zhang and Wijekoon, 2019; Chavan et al., 2020; Woo et al., 2020), the presence of numerous transistors and external bias lines result in relatively high power consumption for the I&F operations. Thus, for energy-efficient neuron circuits, suppression in numbers of transistors, absence of external bias lines, and use of steep switching devices with extremely low subthreshold swings (*SS*s) are needed (Abbott, 1999; Izhikevich, 2003; Cheung, 2010); the steep switching devices are crucially necessary for a substantial reduction in power consumption of neuron circuits.

In this paper, we propose a neuron circuit without external bias lines and verify its I&F operation. The essential component of the circuit is a *p*-*n*-*p*-*n* diode that acts as a steep switching device with an extremely low *SS* for the I&F operation (Moll et al., 1956; Xiaodong et al., 2014). The presence of two terminals in the diode eliminates the need for external bias voltages for the I&F operation, and the low subthreshold current of the diode reduces the power consumption of the neuron circuit. Furthermore, the neuron circuit exhibits temporal integration, triggering threshold, depolarization, repolarization, and refractory period similar to the essential functions of a biological neuron (Bear et al., 2007). In this work, the I&F operation is investigated through the mixed-mode technology computer-aided design (TCAD) simulation (Silvaco, 2016). The firing frequency performance is evaluated by modulating the amplitude and time width of synaptic current pulses flowing into the neuron circuit. The energy consumption, power consumption and energy efficiency are examined and compared with those of a CMOS-based neuron circuit using a digital signal controller.

## Proposed I&F Neuron Circuit

### Device Characteristics

**FIGURE 1:**
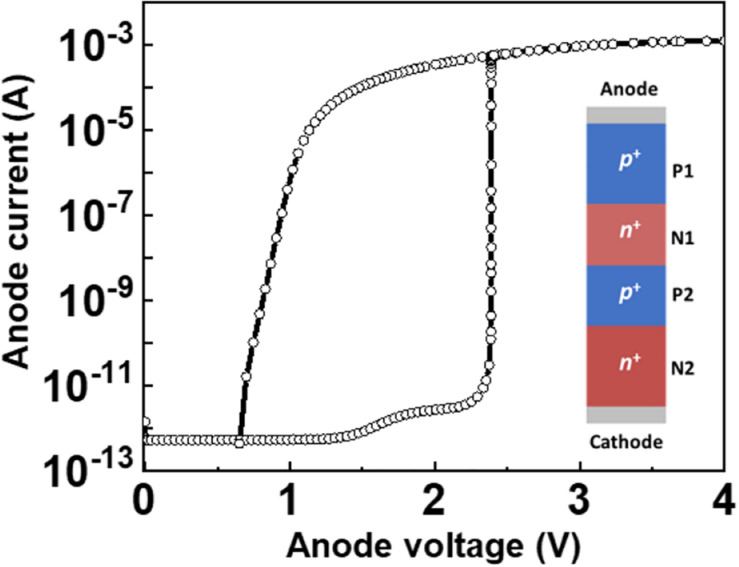
I-V Characteristic of the *p*-*n*-*p*-*n* diode with device schematics.

The dimensional parameters of a diode consisting of a *p*^+^-*n*^+^-*p*^+^-*n*^+^ silicon nanowire (SiNW) are a *p*^+^-doped (P1) region of 100 nm, an *n*^+^-doped (N1) region of 50 nm, a *p*^+^-doped (P2) region of 50 nm, an *n*^+^-doped (N2) region of 100 nm, and a channel thickness of 10 nm (see the inset of Figure 1). The doping concentration are 1 × 10^20^ cm^–3^ for the P1 region, 5 × 10^18^ cm^–3^ for the N1 region, 5 × 10^19^ cm^–3^ for the P2 region, and 1 × 10^20^ cm^–3^ for the N2 region. For the *n*-channel MOSFETs with *n*^+^-*p*^–^-*n*^+^ SiNWs utilized in the neuron circuit without external gate and drain bias, the dimensional parameters are a channel length of 50 nm, an Si channel thickness (*T*_Si_) of 10 nm, and a gate oxide thickness (*T*_O__X_) of 2 nm. Doping concentrations of the *n*^+^-doped source, *p*^–^-doped channel, and *n*^+^-doped drain regions are 1 × 10^20^, 1 × 10^17^, and 1 × 10^20^ cm^–3^, respectively.

Figure 1 shows current-voltage characteristics of the *p-n-p-n* diode in a DC sweep of anode voltage. The *p-n-p-n* diode remains in the off state before the anode voltage (*V*_anode_) is applied. As *V*_anode_ increases from 0.0 to 2.3 V, electrons in the *n*^+^-doped region (N2) and holes in the *p*^+^-doped region (P1) move toward the junction between the *p*^+^-doped region of P2 and the *n*^+^-doped region of N1. Subsequently, reverse bias is formed at the junction, which induces impact ionization with a strong electric field. When the reverse bias exceeds the breakdown voltage of the *p-n-p-n* diode, the diode becomes avalanche breakdown (Holonyak, 2001). The *p-n-p-n* diode has the latch-up properties with a steep subthreshold slope (*SS*) of 0.02 mV/dec and a high on/off current ratio of 10^9^. These characteristics help the neuron circuit to generate the narrow spiking width, thereby providing the low power consumption, high energy efficiency and high firing frequency. In comparison, MOSFETs have a limit of the 60 mV/dec *SS* at 300 K, which is a major obstacle to reduce the operating voltage and power consumption due to the flowing subthreshold current (Lim et al., 2017). Consequently, a neuron circuit using only MOSFETs has the wide spiking width due to the subthreshold current.

### Description and Operation

**FIGURE 2:**
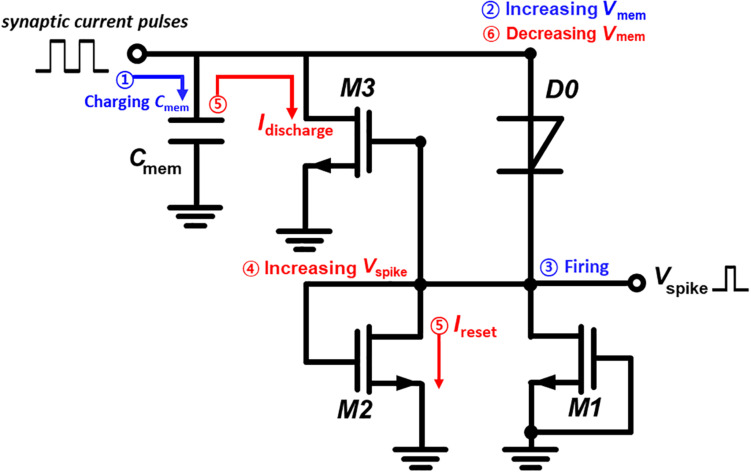
Description and operation mechanism of the presented I&F neuron circuit without external bias lines.

Figure 2 illustrates the construction and operation mechanism of the proposed I&F neuron circuit. The neuron circuit is constructed with one *p*-*n*-*p*-*n* diode (D0), three MOSFETs (M1, M2, and M3), and one membrane capacitor (*C*_mem_). Regarding the roles of the components of the I&F neuron circuit, *C*_mem_ contributes to the increase in the membrane voltage (*V*_mem_); D0 generates spike voltages (*V*_Spike_); M1 acts as a resistor and the resistance contributes to the determination of the *V*_Spike_ value; and M2 and M3 are responsible for resetting the spiking and membrane voltages, respectively. For the operation of the I&F neuron circuit, M1 is in the cut-off mode because of the grounded gate, and M2 is in the saturation mode because of the connection of the gate to the drain; note that the threshold voltages of M1 and M2 are about 0.6 V. Both the resistances of M1 in the cut-off mode and M2 in the saturation mode determine the *V*_Spike_ value by voltage dividing with the diode. Also, the M1, which has constant resistance from the cut-off mode, contributes to restrain unwanted charge-feedthrough caused by capacitive coupling between the M3 channel and its gate (for more details, see Supplementary Material). Most importantly, the presented neuron circuit operation requires no external bias lines.

The I&F operation of the presented neuron circuit begins with the flow of synaptic current (*I*_Synaptic_) pulses from pre-synaptic devices into the neuron circuit. Charges carried by the *I*_Synaptic_ pulses are integrated into *C*_mem_ with a capacitance of 30 pF. The temporal integration of charges increases *V*_mem_ which is the anode voltage of D0. *V*_Spike_ is abruptly generated when *V*_mem_ reaches a triggering threshold voltage of 2.26 V for the latch-up of the anode current of the diode. The *V*_Spike_ value is determined by the voltage division of the diode and M1. The generation of *V*_Spike_ supplies the gate voltages to M2 and M3 and opens the channels of these transistors. The discharge current flows from *C*_mem_ to the ground through the M3 channel, and this flow rapidly decreases *V*_mem_. Simultaneously, the reset current *I*_reset_ flows from the cathode of D0 to the ground through the M2 channel. Eventually, the opening of the M2 and M3 channels resets the anode and cathode voltages to zero, and accordingly *V*_Spike_ becomes zero. Thus, the latch-up of D0 and the subsequent opening of the M2 and M3 channels cause the presented neuron circuit to fire *V*_Spike_ pulses toward post-synaptic devices.

**FIGURE 3:**
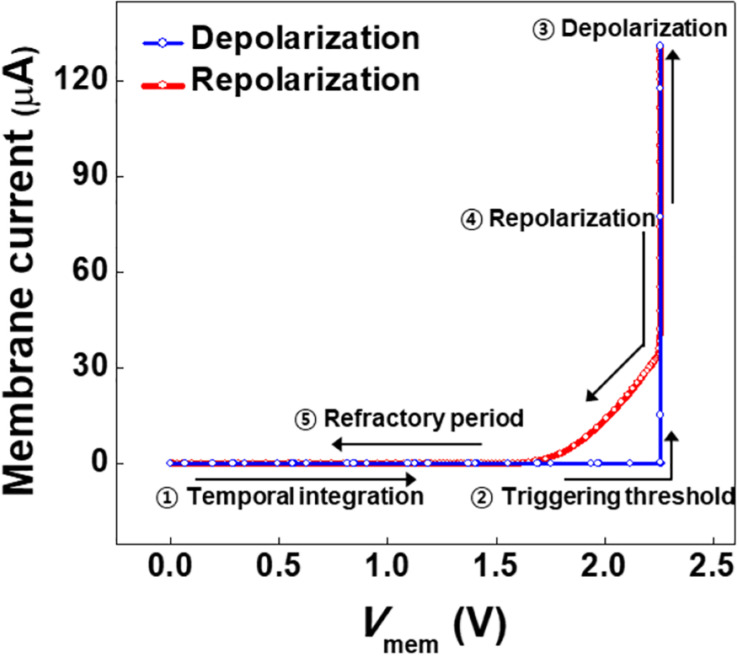
Membrane current as a function of membrane voltage.

Membrane current is the anode current flowing in the channel of D0 in the neuron circuit. The membrane current varies with *V*_mem_ (the anode voltage) as charges are integrated into *C*_mem_ and discharged from *C*_mem_. The membrane current is plotted in Figure 3 as a function of *V*_mem_. The plot demonstrates that the operation of the presented neuron circuit mimics the temporal integration, triggering threshold, depolarization, repolarization, and refractory period of a biological neuron. The membrane current does not flow during the temporal integration of charges in *C*_mem_. When the temporal integration induces a triggering threshold voltage of 2.26 V, the membrane current increases abruptly to 130.9 μA. This abrupt increase in the membrane current in the neuron circuit corresponds to the depolarization of electrical action potential in a biological neuron. The discharging of *C*_mem_ after depolarization leads to a rapid and subsequently gradual decrease in the membrane current, which corresponds to the repolarization of electrical action potential in a biological neuron. As the membrane current becomes negligible, the presented neuron circuit remains in the refractory period.

### Simulation

**FIGURE 4:**
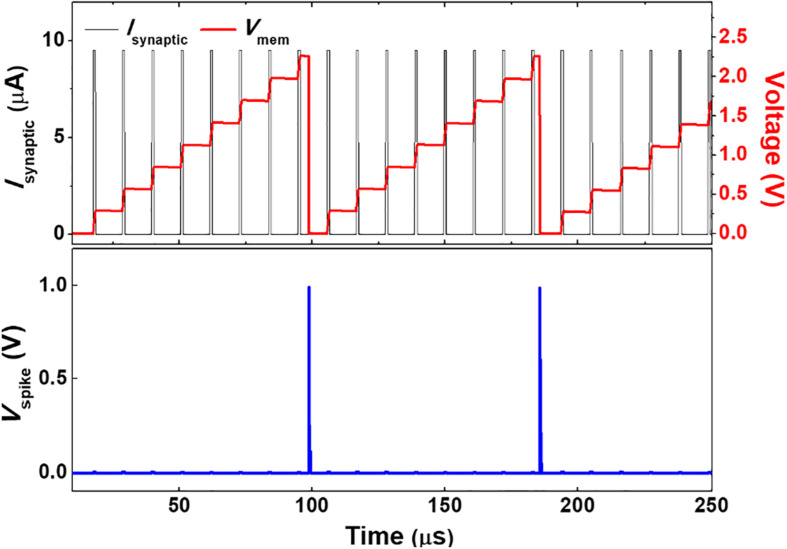
Synaptic current pulses, membrane voltage, and spike voltage pulses of the presented neuron circuit as a function of time.

The synaptic current pulses, membrane voltage, and spike voltage pulses in the I&F operation of the presented neuron circuit are plotted in Figure 4 as a function of time. As the *I*_Synaptic_ pulses with a time width of 0.8 μs and a period of 10 μs are transmitted to the neuron circuit, charges are integrated in *C*_mem_, thereby increasing *V*_mem_. Each *I*_Synaptic_ pulse of 9.5 μA increases *V*_mem_ by 0.28 V during the temporal integration. When *V*_mem_ reaches the triggering threshold voltage of 2.26 V after the arrival of eight *I*_Synaptic_ pulses into *C*_mem_, *V*_Spike_ is generated rapidly from 0.0 to 0.98 V during the depolarization. During the subsequent repolarization, both *V*_mem_ and *V*_Spike_ return to the initial voltage of 0.0 V. Over a single period of the depolarization and repolarization, the presented neuron circuit fires a *V*_Spike_ pulse with an amplitude of 0.98 V. For these *I*_Synaptic_ pulses, the *V*_Spike_ pulse is repeatedly fired at a frequency of 11.5 kHz.

### Dependency of Firing Frequency on Synaptic Current

**FIGURE 5:**
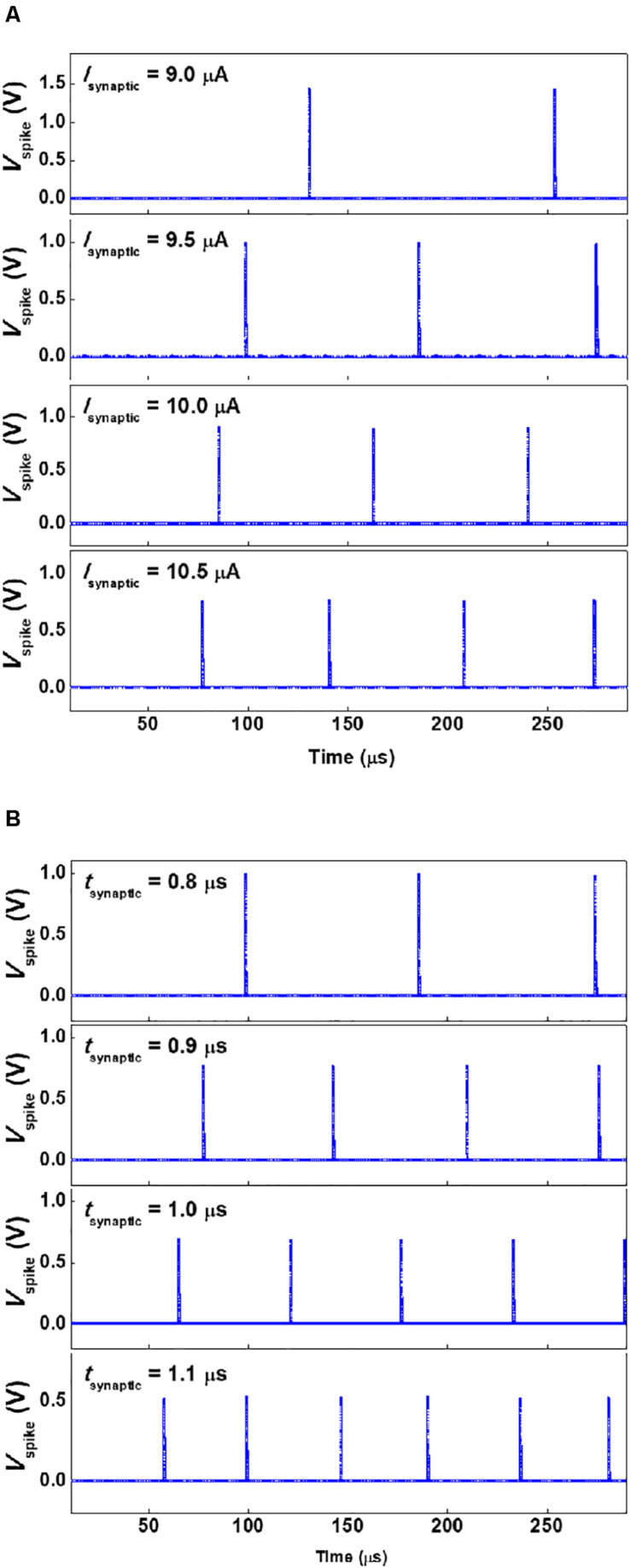
Firing frequency as a function of time for the **(A)** *I*_Synaptic_ amplitudes (with a time width (*t*_Synaptic_) of 0.8 μs and a period of 10 μs) from 9.0 to 10.5 μA by an increment of 0.5 μA, and **(B)** *I*_Synaptic_ time widths (with an amplitude of 9.5 μA and a period of 10 μs) from 0.8 to 1.1 μs by an increment of 0.1 μs.

**FIGURE 6:**
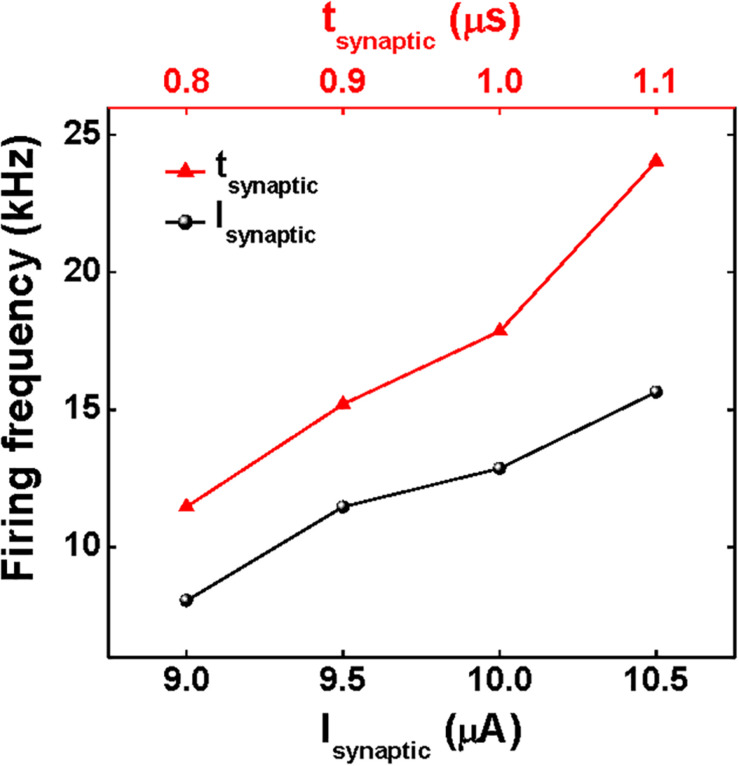
Firing frequency as functions of the time width and amplitude of the *I*_Synaptic_ pulses.

The firing frequency of the presented neuron circuit depends on the amplitude and time width of the *I*_Synaptic_ pulses. This dependency of the firing frequency is depicted in Figures 5, 6. The larger amplitude or the wider time width of the *I*_Synaptic_ pulses decreases the time for *V*_mem_ to reach the triggering threshold voltage. This causes the neuron circuit to fire the *V*_Spike_ pulses at higher frequencies. The firing frequency increases from 8.1 to 15.6 kHz as the amplitude of the *I*_Synaptic_ pulses with a time width of 0.8 μs and a period of 10 μs increases from 9.0 to 10.5 μA by an increment of 0.5 μA. Moreover, the firing frequency shifts from 11.5 to 24.0 kHz as the time width (*t*_Synaptic_) of the *I*_Synaptic_ pulses with an amplitude of 9.5 μA and a period of 10 μs increases from 0.8 to 1.1 μs by an increment of 0.1 μs. The adjustment of the amplitude and time width of the *I*_Synaptic_ pulses control the firing frequency (for more details, see Supplementary Material).

### Superior Energy Efficiency and Power Consumption

**FIGURE 7:**
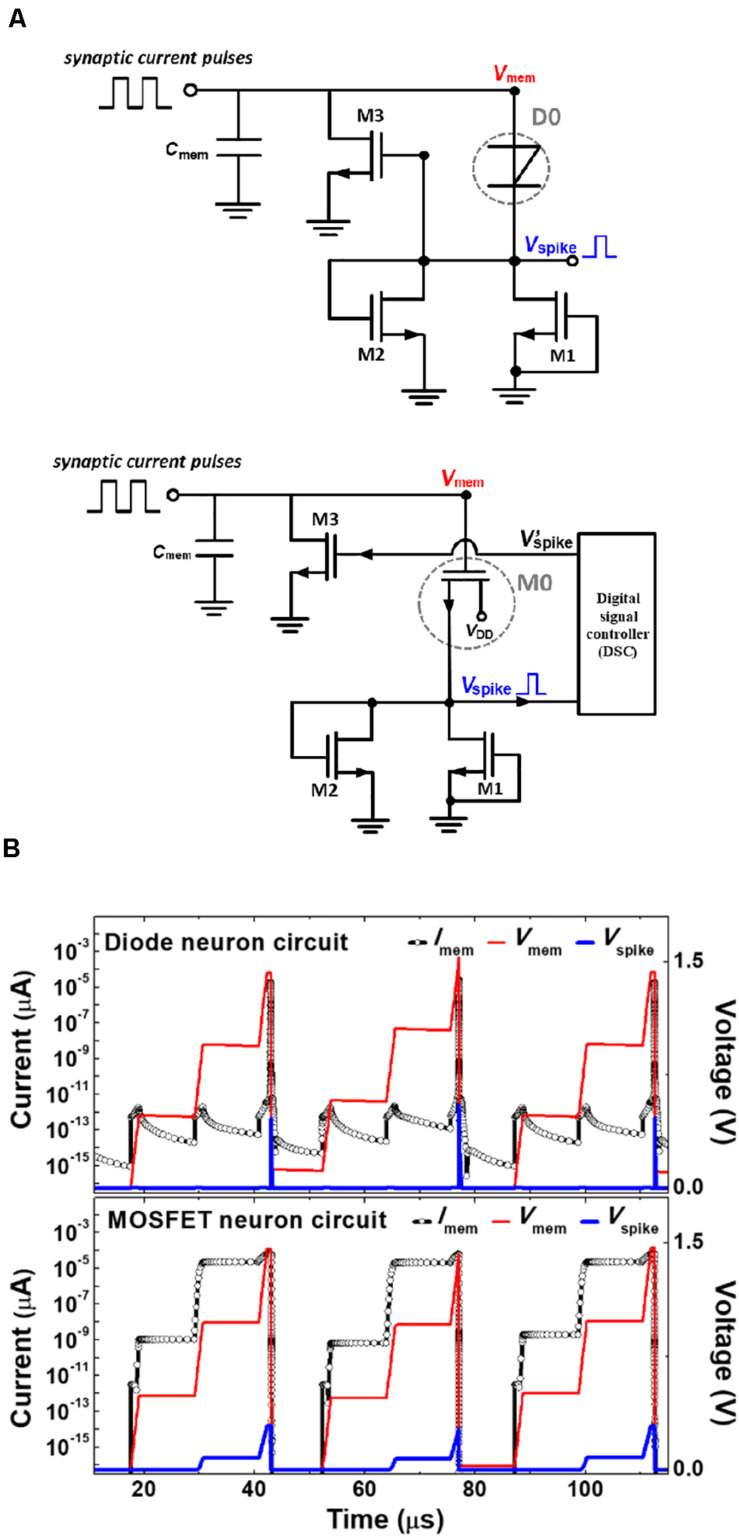
**(A)** Schematic diagrams of the diode neuron circuit and the MOSFET neuron circuit with a digital signal controller, and **(B)** synaptic current pulses, membrane voltage, and spike voltage pulses as a function of time for the diode neuron circuit and the MOSFET neuron circuit.

The *p*-*n*-*p*-*n* diode (D0) utilized in the presented neuron circuit is replaced by a MOSFET (called M0) for the construction of a MOSFET neuron circuit to emphasize the advantages of the use of a *p*-*n*-*p*-*n* diode in the presented neuron circuit over the MOSFET neuron circuit, regarding the energy consumption, power consumption and energy efficiency. The structure and function of the MOSFET neuron circuit are compared with those of the diode neuron circuit in Figure 7. The MOSFET neuron circuit needs a digital signal controller for the I&F operation; the digital signal controller supplies driving pulses to reset the MOSFET neuron circuit when the membrane voltage reaches a threshold voltage of M0. In the MOSFET neuron circuit, *V*_mem_ provides a gate voltage of M0, and the triggering threshold voltage for the opening of the M0 channel is 1.44 V. However, for *V*_mem_ below 1.44 V, *V*_Spike_ is already generated because the *SS* is higher than 60 mV/dec, which forms a greater *V*_Spike_ time width than when the diode is used; this causes the circuit to consume energy more inefficiently while *V*_Spike_ is generated.

For the neuron circuits, the energy consumption, power consumption and energy efficiency are estimated from the following expressions;

(1) $$\mathrm{Energy}\,\mathrm{consumption}\,\left(E\right)=\int_{T}V\,\left(t\right)\times I\,\left(t\right)\,dt\,\left(J\right)$$

(2) $$\mathrm{Power}\,\mathrm{consumption}\,\left(P\right)=\frac{1}{T}\,\int_{T}V\,\left(t\right)\times I\,\left(t\right)\,dt\,\left(W\right)$$

(3) $$\mathrm{Energy}\mathrm{efficiency}\left(\eta \right)=\frac{\begin{array}{c} \int_{T}V\,\left(t\right)\times I\,\left(t\right)\,dt\\ \left(\mathrm{firing}\,\mathrm{period}\,\mathrm{per}\,\mathrm{spike}\right) \end{array}}{\begin{array}{c} \int_{T}V\,\left(t\right)\times I\,\left(t\right)\,dt\\ \left(\mathrm{total}\,\mathrm{period}\,\mathrm{per}\,\mathrm{spike}\right) \end{array}}\times 100\left(\%\right).$$

For a single I&F operation, the MOSFET neuron circuit demands an energy consumption of 0.68 nJ with a power consumption of 19.1 μW and an energy efficiency of 33.0% at *I*_Synaptic_ = 9.5 μA, *t*_Synaptic_ = 1.2 μs, and the firing frequency = 28.1 kHz. In comparison with the MOSFET neuron circuit, the diode neuron circuit consumes much lesser power and energy for a single I&F operation; the energy consumption, power consumption and energy efficiency are 0.59 fJ, 16.7 pW and 96.3%, respectively, under the same conditions of *I*_Synaptic_ as the MOSFET neuron circuit. The excellent power consumption and energy efficiency of the diode neuron circuit originate from the latch-up of the anode current and the high ratio (approximately 10^13^) of the anode current to the off current of the *p-n-p-n* diode. Therefore, the presented neuron circuit utilizing the *p*-*n*-*p*-*n* diode is superior in terms of the power consumption and energy efficiency in comparison with that constructed with only MOSFETs.

### Comparison With Other I&F Neuron Circuits

The diode neuron circuit is compared with other neuron circuits with respect to the device type, and the number of external bias lines, and components needed for I&F operations, as well as energy consumption. In Table 1, the CMOS, floating-gate FET and FBFET neuron circuits reported by other research groups require 5–23 elements with capacitors, and more than 1–10 external bias lines which require extra peripheral circuit for generating bias voltages, causing these neuron circuits to consume high power and energy (Indiveri et al., 2006; Kornijcuk et al., 2016; Choi et al., 2018; Kwon et al., 2018; Kim et al., 2019; Wang and Khan, 2019; Zhang and Wijekoon, 2019; Chavan et al., 2020; Woo et al., 2020). The FBFET neuron circuit has relatively low energy consumption compared to others except ours, but this neuron circuit requires extra peripheral circuits for generating voltage bias and controllers for the I&F operation (Choi et al., 2018; Kwon et al., 2018; Woo et al., 2020). The PDSOI MOS-based neuron circuit also requires an external reset circuit applied with changeable gate voltage to reset the membrane potential (Chavan et al., 2020). In contrast, the presented neuron circuit has only five components and requires no external bias lines. Consequently, this neuron circuit has the lowest energy consumption.

**TABLE 1:** Comparison of the presented neuron circuit with neuron circuits reported by other research groups regarding the based device types, number of external bias lines, and components as well as energy consumption.

	**Neuron model**	**Based device (length/width)**	**Operating mechanism of neuron device**	**Number of external bias lines**	**Approximate components**	**Approximate total energy (J/# of spike)**
Indiveri et al. (2006)	Integrate-and-fire	MOSEFT (0.8 μm/0.8 μm)	Field-effect	7	22 transistors, 1 capacitor	900 × 10^–12^ (at 200 Hz)
Zhang and Wijekoon (2019)	Integrate-and-fire	MOSFET (0.35 μm/0.35 μm)	Field-effect	5	14 transistors, 2 capacitors	9.0 × 10^–12^ (at 1 MHz)
Kornijcuk et al. (2016)	Integrate-and-fire	Floating-Gate FET	FN tunneling	4	13 transistors, 1 capacitor,	1.3 × 10^–12^ (at 23 Hz)
Kwon et al. (2018)	Integrate-and-fire	FBFET	Positive feedback	2	9 transistors 1 resistor, 1 capacitor	8.83 × 10^–12^ (at 500 kHz)
Choi et al. (2018)	Integrate-and-fire	FBFET (1.0μm/0.1 μm)	Positive feedback	3	5 transistors	0.25 × 10^–12^ (at 200 Hz)
Kim et al. (2019)	Integrate-and-fire	MOSFET (0.4 μm/1 μm)	Schmitt trigger	2	6 transistors 1 capacitor,	159 × 10^–12^ (at 1 MHz)
Wang and Khan (2019)	Integrate-and-fire	FEFET (0.08 μm/>0.05 μm)	Ferroelectric field-effect	1	2 transistors, 2 diodes,3 capacitors, 4 resistors	570 × 10^–12^ (at 40 Hz)
Woo et al. (2020)	Integrate-and-fire	FBFET (0.1 μm/–)	Positive feedback	2	4 transistors, 1 capacitor	2.9 × 10^–15^ (at 20 kHz)
Chavan et al. (2020)	Integrate-and-fire	PDSOI MOSFET (0.04 μm/1 μm)	Band-to-band tunneling	3	6 transistors	3.2 × 10^–15^ (at 150 kHz)
This work	Integrate-and-fire	*p-n-p-n* diode (0.05 μm/0.05 μm)	Avalanche breakdown	0	3 transistors, 1 diode, 1 capacitor	5.94 × 10^–16^ (at 28.1 kHz)

## Discussion

The presented neuron circuit consisting of a *p*-*n*-*p*-*n* diode, a capacitor, and three transistors, with an energy consumption of 0.59 fJ and an energy efficiency of 96.3%, does not require any external bias lines for its I&F operation. In the I&F operation, the firing frequency can be adjusted by varying the amplitude and width of the synaptic current pulses. In comparison with

CMOS-, FGFET-, and FBFET-based neuron circuits, the proposed circuit is much simpler, because of the absence of external bias lines and the use of the diode.

Moreover, the presented neuron circuit is superior in terms of the power consumption and energy efficiency in comparison with circuits constructed with only MOSFETs. This research demonstrates the possibility of neuromorphic computing architectures driven by SNNs without external bias lines.

### Simulation Methods

The I&F neuron circuit was carried out with a two-dimensional device simulator (Silvaco Atlas, version 5.20.2 R) (Silvaco, 2016). In the simulation of the *p*-*n*-*p*-*n* diode, the BJT model and Fermi-Dirac statistics were employed to analyze the device characteristics, and the CVT transverse electric-field-dependent mobility of the charge carriers assumed in this work (Han and Choi, 2010). In the MOSFET simulation, the MOS2 model was used for analyzing. The default parameters for these models were used in the simulation.

## Data Availability Statement

All datasets generated for this study are included in the article/Supplementary Material, further inquiries can be directed to the corresponding author/s.

## Author Contributions

Y-SP, SW, and SK: conceptualization and data curation. Y-SP, DL, and SK: methodology. Y-SP and KC: validation and investigation. KC and SK: formal analysis. Y-SP and SK: resources, writing—review and editing, visualization, and project administration. Y-SP and SK: writing—original draft preparation. All authors have read and agreed to the published version of the manuscript and have cooperated in the preparation of this work.

## Conflict of Interest

The authors declare that the research was conducted in the absence of any commercial or financial relationships that could be construed as a potential conflict of interest.
