# Supplementary material for: Integrate-and-Fire Neuron Circuit Without External Bias Voltages
Source: Front Neurosci. 2021 Mar 24;15:644604. doi: 10.3389/fnins.2021.644604 (PMC8024489; doi:10.3389/fnins.2021.644604)
Supplement: Supplementary file 1 [file Data_Sheet_1.pdf]

Supplementary Material

Investigation of wide spectrum of firing frequency

To investigate a spectrum of the firing frequency in the proposed neuron circuit, some additional simulations were performed. As shown in Fig. 6, the neuron circuit seems to have the limitation of the firing frequency ranging from 8.1 to 24.0 kHz. However, if the pre-synaptic current ($I_{synaptic}$) are modulated, the neuron circuit can have a wide spectrum of the firing frequency. For example, even if a time width of 1.0 millisecond and an amplitude of 9.5 μA of the $I_{synaptic}$ pulses are applied unlike the $I_{synaptic}$ pulses used in the main text (the time width = 0.8 μs, the amplitude = 9.5 μA), the integrate-and-fire is still performed. However, it is difficult to visually observe a series of the I&F processes, as shown in Fig. S1 (a). Moreover, because the reset operation is performed while the $I_{synaptic}$ is applied for millisecond, the charge in the membrane capacitor (C_{mem}) cannot be fully discharged while the reset operates. Nevertheless, the limitation of the firing frequency can be overcome by modulating the amplitude of the $I_{synaptic}$ pulses. As shown in Fig. S1 (b), when the amplitude of $I_{synaptic}$ is adjusted to 95 nA, we can observe the integrate-and-fire operation and the fully reset operation.

For the nanoseconds order of the time width of $I_{synaptic}$, the presented neuron circuit generates voltage spikes when the membrane potential (V_{mem}) exceeds threshold point through applied $I_{synaptic}$ pulses without any additional adjustment of the amplitude. In Fig. S1 (c), $I_{synaptic}$ pulses with a wider time width of 8.1 μs is applied to the neuron circuit following the pulses with a nanoseconds width of 10 ns to observe the process of the I&F operation in a time diagram. V_{mem} is increased by C_{mem} charged for nanoseconds of the $I_{synaptic}$ pulses. When V_{mem} exceeds the threshold point, the neuron circuit fires the spike signal. Therefore, the presented neuron circuit has a wide spectrum of the firing frequency obtained by the adjustment of the time width ($t_{synaptic}$) of $I_{synaptic}$.

Diode neuron circuits with and without M1 transistor

Figure S2 shows that synaptic current pulses, membrane voltage, and spike voltage pulses as a function of time for the diode neuron circuits (a) without and (b) with M1 as a function of time. For the diode neuron circuit without M1, it appears that the abrupt application of a current step causes a small and sudden increase in potential on the gate of M2, actually generated by M3 (the gate of M2 is connected to the gate of M3), in particular, "probably" caused by a phenomenon of capacitive coupling between the M3 channel and its gate (a sort of reverse charge-feedthrough). The small increase in the gate voltage on M3 brings it into conduction (subthreshold) sufficiently to discharge the capacitor (V_{mem}); since the gate voltage of M3 is equal to M2, it is brought into conduction in the subthreshold regime also M2 (which is also in saturation), during these exponentially discharged bumps. In contrast, for the diode neuron circuit with M1, the presence of an "equivalent" channel resistance due to M1 (in the cut-off region), discharges the charges faster reducing the amplitude of the voltage peak due to the capacitive coupling so that the voltage peak is small, not large enough to make conductive (in the subthreshold region) M2 and M3 during the integration of synaptic current pulses.

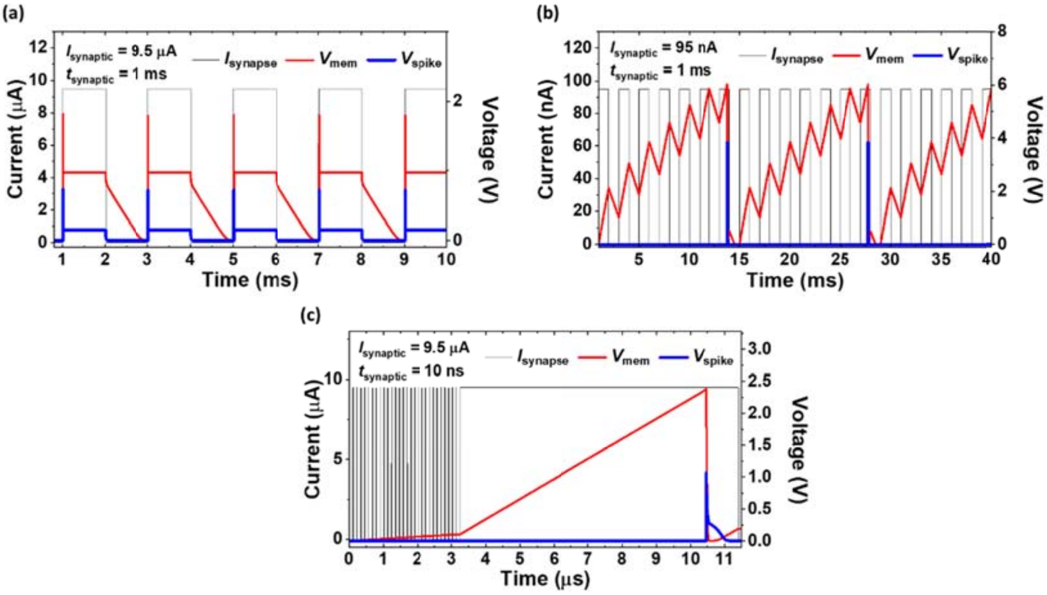

Figure S1. Integrate-and-fire operation in the order of the milliseconds time width ($t_{synaptic}$) of 1 ms (a) with the amplitude of 9.5 μA and (b) 95 nA, and (c) in the order of nanoseconds with the amplitude of 9.5 μA.

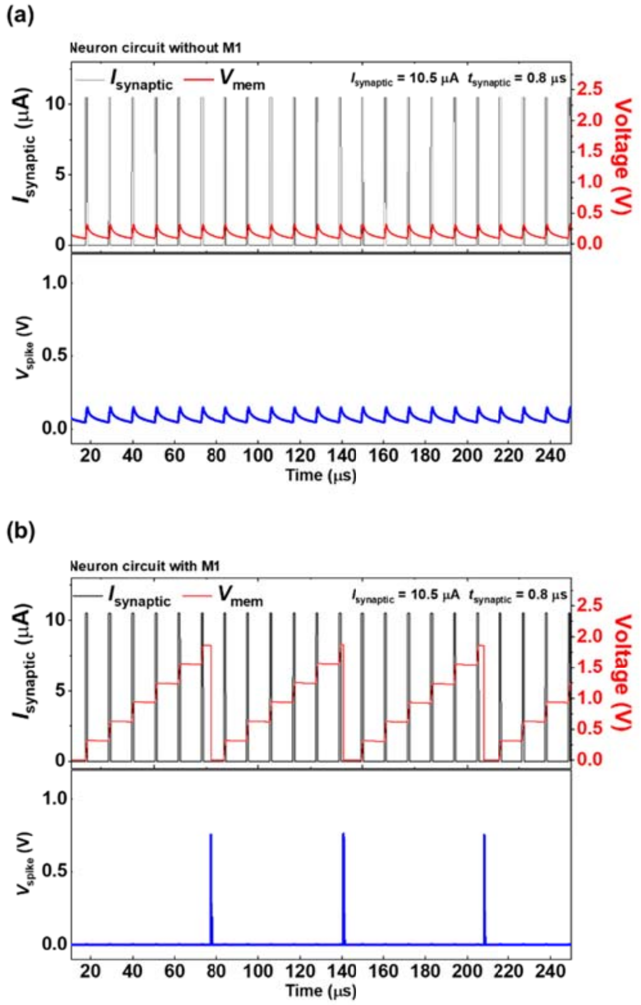

Figure S2. Synaptic current pulses, membrane voltage, and spike voltage pulses of diode neuron circuits (a) without and (b) with M1 transistor as a function of time.
